# Substance-induced anxiety disorder after one dose of 3,4-methylenedioxymethamphetamine: a case report 

**DOI:** 10.1186/s13256-018-1670-7

**Published:** 2018-05-25

**Authors:** Kaeley Kaplan, Fiona Kurtz, Kelly Serafini

**Affiliations:** 1 Swedish First Hill Family Medicine Residency, Swedish Medical Center, 1401 Madison Street, Suite 100, Seattle, WA 98104 USA; 2 Department of Clinical Psychology, Seattle Pacific University, Seattle, WA USA

**Keywords:** Substance-induced anxiety disorder, 3,4-Methylenedioxymethamphetamine, MDMA, Selective serotonin reuptake inhibitor, Mental health, Primary care

## Abstract

**Background:**

In this report, we describe a case of a patient with substance-induced anxiety disorder occurring after a single dose of 3,4-methylenedioxymethamphetamine. Furthermore, we describe the use and efficacy of the Primary Care Behavioral Health model, a collaborative approach to integrative primary mental health care, in evaluating and treating this rare mental health disorder.

**Case presentation:**

Three days following ingestion of one dose of 3,4-methylenedioxymethamphetamine, a 35-year-old Hispanic man with no significant prior mental health history and no history of prior 3,4-methylenedioxymethamphetamine use presented to our hospital with severe, acute anxiety and panic symptoms. He was initially treated with a combination of behavioral therapy and the serotonin agonist buspirone. Buspirone ultimately proved ineffective, so it was discontinued in favor of the selective serotonin reuptake inhibitor sertraline. While awaiting the pharmacological onset of sertraline, the patient worked with a behavioral health consultant, who provided psychoeducation on the experience of panic, building relaxation skills, and modifying maladaptive thought patterns. Enhanced communication between the primary care provider and behavioral health consultant facilitated the planning and enactment of the patient’s care plan. Approximately 2.5 months after his initial ingestion of 3,4-methylenedioxymethamphetamine, the patient’s symptoms subsided. This improvement was attributed to the combination of the behavioral health intervention and sertraline at a dose of 50 mg daily. Six months after 3,4-methylenedioxymethamphetamine ingestion, the patient began to gradually taper sertraline and has had no resurgence of anxiety symptoms to date.

**Conclusions:**

Our patient’s case not only demonstrates a rare presentation of 3,4-methylenedioxymethamphetamine-induced anxiety disorder but also provides support for the use of the Primary Care Behavioral Health model to deliver individualized, timely mental health care in a primary care setting.

## Background

3,4-Methylenedioxymethamphetamine (MDMA), more commonly known as “molly” in its pure form and as “ecstasy” historically, is a drug that gained popularity in the 1980s and continues to be used around the world [1, 2]. Current data suggest that MDMA use is prevalent in the United States. According to the 2014 National Survey on Drug Use and Health, approximately 609,000 people over the age of 12 years had used MDMA in the previous month, and among adults aged 26 years or older, 6.5% reported lifetime use of MDMA, and 0.1% reported MDMA use in the previous month [2]. Recreational users have reported that the drug induces “feelings of intimacy and closeness to others” and “positive mood” [3, 4]. Acute (< 1 day) and subacute (1–7 days) negative effects of the drug include confusion/delirium, loss of appetite, insomnia, panic/anxiety, low mood/depression, and difficulty concentrating, with low mood typically occurring 3–4 days after MDMA use [4]. The longer-term psychological effects of MDMA are more difficult to define, in part because many MDMA users are polydrug users and in part because of the high prevalence of comorbid disorders such as depression, anxiety, and insomnia [1]. Even less is known about the possible long-term effects of a one-time dose of MDMA, though several case reports, including the present one, highlight lasting effects after a single dose [5, 6].

The drug’s chief psychopharmacological target is the monoaminergic system, which plays a role in emotional regulation and physiological arousal [1, 7]. Research in rodents and nonhuman primates has consistently demonstrated an MDMA-triggered release of dopamine, norepinephrine, and serotonin from nerve terminals [7, 8]. Repeated doses of MDMA lead to damage of serotonergic nerve terminals, perhaps via increasing free radical activity, and there is concern and some clinical evidence for long-term depletion of serotonin and changes to the serotonergic neural network in heavy users of MDMA [1, 7]. Alterations in serotonergic activity have long been implicated in anxiety disorders, and feelings of panic/hyperarousal are induced by increased activity of epinephrine and norepinephrine, adding strength to the hypothesis that MDMA use can elicit anxiety and panic symptoms [9].

Patients’ concerns regarding illicit substance use (e.g., MDMA use) and mental health are increasingly addressed and treated in the primary care setting. Primary care facilities provide an easily accessible and comfortable space to discuss a wide range of medical and psychiatric concerns [10]. In fact, 50–70% of people experiencing psychological or behavioral conditions receive their treatment in primary care settings from primary care providers (PCPs) [11, 12], and 80% of individuals with a mental health disorder report that they will visit their PCP at least one time within a calendar year [13]. Thus, PCPs are the most likely health care providers to assess and treat substance use concerns.

Recently, some pioneering medical clinics have begun to adopt a Primary Care Behavioral Health (PCBH) model with the aim of strengthening integration between physical and mental health care. This approach is particularly applicable to and beneficial in treating patients with depression, anxiety, posttraumatic stress disorder, and substance use disorder symptoms or concerns. These patients often benefit from both psychopharmacological and behavioral health treatment. The PCBH model has a population-based health focus and aims to meet the behavioral health needs of all patients receiving medical treatment in primary care, including those who are more likely to experience barriers to specialty mental health care because of cost, language differences, stigma, and transportation difficulties [14]. Arguably one of the greatest benefits of this integrated model is strengthened communication between mental health providers (commonly referred to as *behavioral health consultants*) and medical care providers [14]. This enhanced communication facilitates unification of psychopharmacological and behavioral health interventions and maximizes opportunities for PCPs to receive treatment feedback, particularly with unique cases. Given the novelty and complexity of substance-induced psychiatric disorders, patients may benefit greatly from the high level of communication and coordination afforded by the PCBH model.

In this case report, we describe a patient who took a single dose of MDMA that triggered substance-induced anxiety disorder which lasted for several months. The patient’s symptoms gradually improved and remitted with a combination of medical and behavioral therapy in a primary care office that had adopted principles of the PCBH model. The PCP and behavioral health consultant worked closely together and with the patient in providing comprehensive mental health care. This case report highlights both an uncommon presentation of substance-induced anxiety disorder and a unique approach to integrative mental health treatment.

## Case presentation

A previously healthy 35-year-old Hispanic man with a remote history of mild performance anxiety in late adolescence presented to our family medicine residency clinic to establish and seek care for acute onset of anxiety. The patient was a manager at a large manufacturing firm and had received a master’s degree. At the time of presentation, he was married with two children. He had no family history of mental illness and was not taking prescription medications. At his initial visit at the clinic, he reported that 9 days prior, he had taken one dose of “molly” while at a gathering with friends. The friend who had supplied the drug stated that it was “pure crystal MDMA.” According to the patient, this was his first lifetime use of MDMA. He had also consumed several alcoholic drinks that night, reportedly reaching the level of intoxication. He described having a “fine” experience with the drug and returned to his normal baseline for the next 2 days. On the third day after ingesting MDMA, he began to experience an increase in worry and agitation; he reported having panicked thoughts and development of palpitations, blurry vision, flushing, increased thirst, and insomnia. He stated that these symptoms increased over the coming days, prompting him to seek medical care. Regarding substance use history, the patient reported occasional social alcohol use since his early 20s, rarely to excess. He had used cannabis several times while in college (ages 18–22) and found that this precipitated anxiety and therefore he did not continue using it regularly. He denied any regular use of other illicit substances.

On the initial day that the patient met with a medical provider in the clinic, he reported a score of 20 (maximum score of 21) on the Generalized Anxiety Disorder 7-item scale [15], an anxiety screening and rating tool commonly used in primary care offices. This score was consistent with a severe level of anxiety. The patient was prescribed buspirone, a serotonin 1A receptor (5-HT1A) receptor agonist, with a plan to uptitrate to 15 mg twice daily over the coming weeks. The patient was also referred to the clinic’s behavioral health service for adjunctive treatment and was promptly seen by a behavioral health consultant for their first session the following day. This initial visit involved an assessment of his biopsychosocial history and mood, a functional analysis of his anxiety symptoms, and a collaborative discussion regarding his treatment goals. Using interventions informed by cognitive behavioral therapy, the patient and the behavioral health consultant aimed to increase his coping skills and management of his anxiety symptoms and to improve his overall quality of life (e.g., reduce distress, increase enjoyment at home, and increase productivity at work).

The patient was seen for follow-up every 7–10 days by his PCP for the next month (*see* Table 1). Simultaneously, he received behavioral health treatment each week following his first month of medical treatment in the family medicine clinic. He then established a therapeutic relationship with a counselor outside the clinic, where they reportedly engaged in talk therapy weekly. The patient reported only a slight improvement in his anxiety and panic symptoms despite the therapeutic dose buspirone; therefore, he was prescribed a selective serotonin reuptake inhibitor (SSRI) and a benzodiazepine and was referred to the psychiatry department for additional consultation. The consulting psychiatrist was concerned for MDMA-induced anxiety disorder and recommended discontinuation of buspirone and initiation of low-dose sertraline with slow uptitration. The patient benefited from behavioral health treatment specifically aimed at enhancing understanding and controlling the sympathetic nervous system (i.e., cognitive behavioral modeling, psychoeducation on the cycle of panic, and relaxation skill training). Behavioral health treatment was especially important as he awaited the clinical effect of his psychoactive medications.

**Table 1:** Patient presentation and treatment timeline

Day 0	Patient reports having ingested MDMA
Day 3	Development of acute anxiety and physiological arousal
Day 9	Patient presents to clinic for evaluation and is prescribed uptitration of buspirone
Week 2	Patient is prescribed lorazepam
Week 3	Patient is taking full dose of buspirone with no significant improvement in anxiety; rare lorazepam use Prescribed SSRI but does not start taking First visit with behavioral health consultant, total of six visits every 1–4 weeks
Week 4	Seen by psychiatrist and given tentative diagnosis of substance-induced anxiety disorder SSRI recommended
Week 6	Begins SSRI sertraline and begins buspirone taper
Week 7	Reports some improvement in anxiety and sleep
Week 7.5	Reports acute worsening of anxiety with suicidal ideation Sertraline dose is lowered, and in 3 days, patient no longer has suicidal ideation
Weeks 8–10	Continues sertraline, reaching dose of 50 mg once daily with continued improvement in anxiety
Weeks 10+	Seen by psychiatrist 2, who concurs with diagnosis of substance-induced anxiety disorder Patient continues sertraline 50 mg/day for several more months without resurgence of anxiety symptoms

*MDMA* 3,4-Methylenedioxymethamphetamine, *SSRI* Selective serotonin reuptake inhibitor

Ongoing evaluation by the PCP, a consulting psychiatrist, and the behavioral health consultant supported a diagnosis of substance-induced anxiety disorder. He experienced persistent anxiety (reporting daily worry, panic, racing heart, dizziness, restlessness, and catastrophic thinking), and all of his symptoms developed shortly after ingesting a single dose of MDMA. His symptoms caused him significant distress and impairment in his employment as well as his family life. The patient denied clinically significant anxiety directly prior to taking MDMA; his only history of anxiety was performance anxiety many years prior. Therefore, he did not meet criteria for panic disorder or generalized anxiety disorder, because his symptom onset followed substance ingestion. Simultaneously, the patient reported transdiagnostic depressive symptoms, including hopelessness, fatigue, maladaptive thinking, and low mood. These symptoms, which also began following the patient’s use of MDMA, were etiologically attributed to his anxious physiological symptoms and thoughts, in particular the catastrophic and generalized worries that this one-time drug use had “ruined” his life. Consequently, his symptoms appeared to be explained by the substance-induced anxiety, as opposed to representing a discrete depressive disorder.

The patient initially tolerated the sertraline well and experienced a relatively rapid improvement in anxiety symptoms while taking 25 mg daily. After 8 days of the 25-mg dose, the dose was increased to 37.5 mg. After 2 days at this dose, the patient developed abrupt onset of suicidal ideation with a resurgence of anxiety and panic symptoms. Given the gravity of these new symptoms, the PCP and behavioral health consultant worked together and with the patient to devise a plan for ongoing care. He was able to see the behavioral health consultant for an urgent visit. The dose of sertraline was reduced to 25 mg, and plans were made for intensive outpatient mental health treatment at a nearby hospital.

While awaiting entrance into that program, his suicidal ideation and anxiety abated. As his symptoms improved, the behavioral health consultant supplemented sympathetic nervous system training with thought identification and cognitive retraining. These interventions served to address his reported catastrophic and demoralizing appraisals following the use of MDMA (e.g., “I am a terrible person for taking that Molly,” and “I have ruined my life forever”). He saw a second psychiatrist for additional recommendations. They concurred with the diagnosis of substance-induced anxiety disorder and the prescribed SSRI treatment. Eventually, the dose of sertraline was slowly increased to 50 mg with continued improvement in all symptoms and no further resurgence of suicidal ideation. The patient’s anxiety and panic were not well controlled until approximately 2.5 months after ingesting MDMA. At 6 months following his presentation, he was doing well with a plan to slowly taper the sertraline. He expressed gratitude for an interdisciplinary team approach and the unique benefits of skills training in tandem with psychopharmacological treatment.

## Discussion

In our patient, a single dose of MDMA triggered anxiety that lasted nearly 2.5 months and eventually improved with the implementation of both psychopharmacological and behavioral health techniques. We know of only one other case report in which one dose of MDMA was reported to precipitate protracted anxiety and/or panic symptoms [5], though given the general underreporting of illicit substance use, the true number may be higher. Clinicians should consider recent MDMA use as a potential etiology of acute anxiety.

Substance-induced mental health disorders are challenging not only to diagnose but also to treat, particularly given the complex nature of substance interactions and overall patient presentation. In our patient, the diagnosis of substance-induced anxiety disorder was corroborated by three separate practitioners (a behavioral health consultant and two independent psychiatrists). The patient had denied any significant precipitants that may have triggered his anxiety, and there was a consistent temporal relationship between MDMA ingestion and his symptoms. Although the treating providers had no reason to doubt the patient’s stated history of one-time MDMA use, it is possible that he unknowingly had ingested other or additional substances. The treating providers did not employ a urine drug screen during the course of the patient’s evaluation and treatment, chiefly because few substances can be reliably detected 9 days postingestion. Therefore, the patient’s MDMA use must be taken at face value as the cause of his symptoms.

Medically speaking, initial treatment with the 5-HT1A receptor agonist buspirone was not effective in relieving the patient’s anxiety and should have been reserved as an adjunctive treatment to an SSRI-based treatment regimen, not as sole therapy. Offering a benzodiazepine at the first visit may have helped the patient more quickly gain control of his anxiety (it was not prescribed until the second visit), though benzodiazepines can interfere with cognitive behavioral therapy and should be deployed with caution owing to concerns surrounding dependence and abuse. It was not until uptitration of the SSRI sertraline that the patient began to experience consistent symptomatic improvement. Notably, this patient did experience a brief worsening of his anxiety while initiating sertraline; this is a relatively common and well-known side effect of SSRIs. In this case, the patient’s acute worsening of symptoms lasted only a few days. Many psychotropic therapies take weeks to months to take effect, and a key component of the patient’s care was timely access to mental health care that served to alleviate symptoms while awaiting the therapeutic effect of medications.

The PCBH model served to offer collaborative, interdisciplinary care that treated both his short-term and long-term needs. The patient’s acute needs were met by psychopharmacological treatment aimed at reducing his debilitating physiological panic and anxiety symptoms. The providers were able to offer long-term benefits by tailoring a behavioral health treatment plan that helped the patient modify newly exacerbated maladaptive thought patterns (cognitive retraining) as well as develop long-term behavioral skills to manage his physiological symptoms (relaxation training).

His overall treatment was bolstered by adherence to the PCBH model’s recommendation of frequent, efficient, and consistent communication between multidisciplinary team members [14]. Use of this approach prevented delays in the patient’s treatment and enhanced treatment plan development and agreement among team members. The PCBH model has been found to be effective for treating patients across the entire spectrum of mental health conditions [10]. The present case report corroborates such findings and demonstrates the feasibility and benefits of integrating behavioral and primary medical care [14]. The PCBH model offers the advantage of combining the resources and knowledge of multiple providers in a single, unified, and accessible setting.

## Conclusions

This report describes a rare presentation of substance-induced anxiety disorder after ingestion of a single dose of MDMA. The patient’s symptoms were severe and were addressed with a combination of behavioral health and psychopharmacological therapies. His symptoms stabilized and improved only after several months of behavioral health counseling and medical treatment with the SSRI sertraline. We posit that his care was augmented by the direct integration of his medical treatment with his behavioral health treatment according to the PCBH model. This integration allowed for timely communication between the patient’s behavioral health provider and PCP and facilitated the assessment and treatment of his unique mental health disorder.
